# Legal Notes for Institutional Workers

**Published:** 1911-10-07

**Authors:** 


### LEGAL NOTES FOR INSTITUTIONAL WORKERS.

The Editor will be pleased to answer legal queries in this column.

### HOSPITAL ABUSE.

Can the miserable person who abuses the hospital be made amenable to the law? We have all heard of the lady who, after a visit to the out-patients' department, sent the hall porter round the corner to fetch her carriage. Was she not guilty of obtaining something by false pretences? Where imposition is suspected, inquiry is generally made as to the means of the patient, and it is clear that a misleading or an untruthful answer would be sufficient to justify a charge of fraud. Cases of that class are comparatively easy to deal with. Assume, however, that a patient having £1,000 a year in the Funds comes to hospital dressed in rags. He is asked no question as to his means, and he volunteers nothing. The only engine of the law which could possibly be set in motion is that which penalises one who obtains "any chattel, money, or valuable security" by false pretences. Where mere advice or treatment is the sole result of the visit, there appears to be no criminal offence. But if pill or potion were supplied, it might be a chattel obtained by "false pretences." But what is a false pretence? That it may be inferred from acts is clear. Thus it was held that a "townee" at Oxford who went to a shop arrayed in cap and gown and obtained goods on credit was guilty of obtaining them by false pretences. Would a jury be entitled to say that every person who goes to the out-patient department impliedly represents that he or she has no means wherewith to pay a medical attendant? While this is a question for a bench of judges, we have little doubt that, if the facts of such a case could be brought before a jury, it would be prepared to find that a fraud had been committed. At any rate, it would be very interesting to see the result of a prosecution instituted against the particularly mean type of person who battens on the hospital and defrauds the doctor of his well-earned fees.

### TO AVOID STRIKES.

Recent events have shown that the "strike universal" is not wholly outside the range of practical politics. Whatever the cause, the feeling of unrest is in the air; and those who are responsible for the smooth working of hospitals and similar institutions cannot afford to ignore the fact. The medical staff is not likely to strike for higher pay, the salary of most hospital doctors being such that if it were doubled it would not be increased! Nor need the loyalty of the nursing staff be feared; but when the strike fever is so rampant that it actually extends to school children, who can say that the domestic staff at a hospital may not be one day infected? To ensure peace be prepared for war, is an old maxim. One method of preventing the sudden strike was explained the other day before the Royal Commission. Each servant when taken on was required to procure a surety for his good behaviour. The security was to be mulcted in the sum of £20 if the servant left his employment without notice. No strike took place where this system was in vogue! Should there be any real fear of a strike among the lower staff of a hospital, it ought not to be difficult to obtain securities for the good behaviour of each new servant employed.

### MR. TROUTBECK AND MIDWIFERY.

While it is the primary function of the Coroner to assist his jury to investigate the cause of death, some of these officials never seem to miss an opportunity of descanting upon the shortcomings of authorities and persons who are away from the court and have no opportunity of being heard on their defence. If a Coroner's homily is based upon an accurate statement of the law he is within his right; but, as Disraeli once said, "It is easier to be critical than to be correct." At an inquest held at Lambeth last Saturday Mr. Troutbeck took occasion to pass some strictures upon the authorities at Guy's Hospital for allowing a nurse who was not a registered midwife to attend a confinement. A child was taken, on the instruction of the nurse in attendance, to Guy's Hospital, where a doctor prescribed some tablets and directed that the infant should be taken home. It subsequently died, the cause of death being suffocation from bronchitis, said by Dr. Freyberger to have been produced at birth. The mother had been attended by a trained hospital nurse, who had been at Guy's for six weeks as a pupil midwife and had attended twenty-two births. She was assisted by another pupil midwife. The Coroner said it was surprising that cases should be attended by nurses from a hospital who, however competent they might be, were not on the register. He founded this remark upon the following statement of the law, which we take from a note in the *Times*, October 2: "It is distinctly contrary to the Midwives Act, which laid it down that persons who were not doctors must not attend cases professionally unless they were registered." Even if this were an accurate pronouncement (which it is not), we should like to hear what the hospital authorities have to say before judging of the merits; but, if he is accurately and fully reported, the Coroner was wrong. The Midwives Act only prohibits unregistered practice "habitually and for gain, except under the direction of a qualified medical practitioner." Was this nurse practising for gain? Moreover, the restriction does not apply to anyone rendering assistance in a case of emergency. The report which we have seen says nothing about emergency. It is satisfactory to note that the jury absolved the nurse from blame; but of course, taking the law from the Coroner, they expressed the opinion that the hospital authorities should send a certified nurse to such cases.

